# ELEVATED CA 19-9 IN AN ASYMPTOMATIC PATIENT: WHAT DOES IT MEAN?

**DOI:** 10.1590/0102-672020220002e1687

**Published:** 2022-09-16

**Authors:** José Donizeti de MEIRA-JÚNIOR, Thiago Nogueira COSTA, Andre Luis MONTAGNINI, Sergio Carlos NAHAS, Jose JUKEMURA

**Affiliations:** 1 Universidade de São Paulo, Medical School, University Hospital, Department of Gastroenterology, Digestive Surgery Division - São Paulo (SP), Brazil.

**Keywords:** CA-19-9 Antigen, Pancreas, Pancreatic Neoplasms, Gastrointestinal Diseases, Antígeno CA-19-9, Pâncreas, Neoplasias Pancreáticas, Gastroenteropatias

## INTRODUCTION

Carbohydrate antigen 19-9 (CA 19-9), first described in 1979, is a cell surface glycoprotein complex produced by ductal cells in the pancreas, biliary system, and epithelial cells in the stomach, colon, uterus, and salivary glands^19^. Its expression is only observed in patients with Lewis antigen (Le) A−B+ or Le A+B− blood groups. Up to 6% of the Caucasian and 22% of the non-Caucasian population are genotypically Le A−B− and therefore do not produce CA 19-9^19^.

CA 19-9 is overexpressed in many benign and malignant, gastrointestinal, and extra-gastrointestinal diseases. Its main implications are in pancreatic ductal adenocarcinoma and intraductal papillary mucinous neoplasm (IPMN), but it can also be elevated in biliary, hepatocellular, gastrointestinal, urological, pulmonary, gynecological, thyroid, and salivary gland cancers^16^. Benign conditions in which CA 19-9 may be elevated include pancreatitis, pancreatic cysts, diabetes mellitus, liver fibrosis, benign cholestatic diseases, and other urological, pulmonary, and gynecological diseases^15^.

The aim of this article was to present a case of an asymptomatic and exuberant elevation of the CA 19-9 with no identified etiology and a review of the clinical use and implications of the CA 19-9.

### Case Report

**Figure 1 -:**
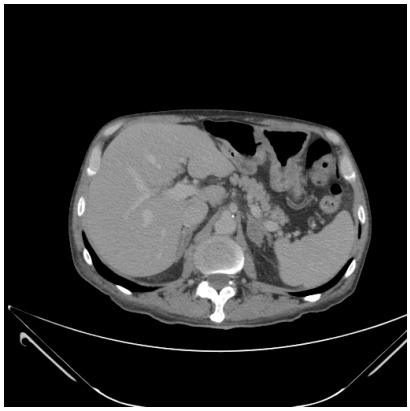
Abdominal computed tomography showing a normal image of the pancreas.

**Figure 2 -:**
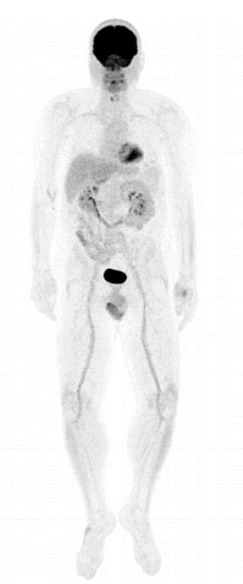
PET-CT showing normal glycolytic metabolism.

A 52-year-old male patient presented for gastroenterological consultation due to an asymptomatic CA 19-9 elevation discovered in routine laboratory testing. The patient denied any gastrointestinal complaints, had no history of previous or current abdominal pain, jaundice, pruritus, fever, or any other biliary disease. He had gained 0.5 kg during the pandemic period. His general practitioner had ordered serum CA 19-9 as a routine laboratory testing. The first test result was 96,544.3 U/mL. Then, he was submitted to an abdominal computed tomography (CT) scan (Figure 1), magnetic resonance imaging with cholangiopancreatography, and positron emission tomography (PET)-CT (Figure 2) sequentially, which did not show any abnormalities. An endoscopic ultrasound showed minimal dilatation of the ventral pancreatic duct near the papilla without any lesion.

As no etiology for this raised CA 19-9 was found, it was decided to follow the patient with repeated dosing of the tumor marker. One month after the first test, the CA 19-9 was 2822 U/mL; in the following month, 343 U/mL; and in the month after, 48.3 U/mL. The final result was 20.8 U/mL.

## DISCUSSION

CA 19-9 is widely used as a tumor marker related to pancreatic ductal adenocarcinoma. Pancreatic cancer (PC) is the fourth leading cause of cancer deaths worldwide, with a 5-year survival rate of less than 7%^2^ ^,^ ^21^. CA 19-9 levels are elevated in more than 80% of patients with advanced PC^12^. Nevertheless, for diagnostic purposes, most international guidelines recommend using CA 19-9 in combination with radiological investigations, such as pancreas protocol CT, which is the current gold standard^1^ ^,^ ^7^. Threshold levels for the elevated CA 19-9 regarding its diagnostic value in PC were established at >37-40 U/mL by a systematic review^9^, which reported sensitivity of 79.0%, specificity of 82.0%, positive predictive value of 72.0%, and negative predictive value of 81.0%.

Up to 10-50% of benign pancreatic diseases (e.g., pancreatitis) and pre-malignant lesions (e.g., IPMNs) have increased CA 19-9 levels (16). Therefore, CA 19-9 levels alone cannot differentiate these from true PCs. This is one of the reasons why CA 19-9 should not be used as a screening tool for PC. CA 19-9 has limited screening utility even in high-risk populations, such as patients with familial PC or Peutz-Jeghers syndrome, with normal results even when imaging revealed preinvasive lesions^19^. Therefore, CA 19-9 plays no role in PC screening in asymptomatic individuals.

In contrast, once the diagnosis of PC is confirmed, CA 19-9 levels are extremely important for proper staging and treatment definition. Preoperative CA 19-9 levels are associated with PC prognosis^4^ ^,^ ^8^. Currently, biological staging of PC is considered for treatment planning, and patients with anatomically resectable PC but with CA 19-9 higher than 500 IU/mL could benefit from neoadjuvant therapy^13^. This cutoff comes from a large study which demonstrated that in patients with preoperative CA 19-9 levels higher than 500 IU/ml, the resectability ratio was less than 70% and the median survival time after resection was less than 20 months^11^.

After the surgery, following the post-resection CA 19-9 levels prior to the initiation of adjuvant chemotherapy is an important prognostic tool and can indicate response to therapy^10^ ^,^ ^17^. In the follow-up after surgical and adjuvant treatment, CA 19-9 elevations have been shown to precede clinical/radiological recurrence by up to 6 months^3^.

Regarding IPMNs, a pre-malignant pancreatic condition, serum CA 19-9 is considered a “worrisome feature” when raised^23^. It is important to consider that the CA 19-9 alone is ineffective in distinguishing malignant pancreatic cysts but useful when associated with other characteristics, such as imaging or cyst size >3 cm^6^. Serum CA 19-9 levels >37 U/mL are a relative indication for IPMN resection according to the European evidence-based guidelines on pancreatic cystic neoplasms^14^. Cyst fluid CA 19-9 obtained from endoscopic ultrasonography-guided fine-needle aspiration is less accurate than other cyst fluid tumor markers such as CEA and CA 125 in differentiating between different pancreatic cystic lesions^16^.

CA 19-9 plays an important role in the diagnosis, staging, and follow-up of cholangiocarcinoma. CA 19-9 above 100 U/mL on a biliary stricture with malignant imaging features suggests perihilar colangiocarcinoma^5^. CA 19-9 also correlates with the prognosis of gastric cancer, including tumor stage, vascular invasion, and lymph node and distant metastasis^22^. CA 19-9 levels alone are not recommended for staging gastric cancer but should be used in combination with CEA and CA 72-4^20^.

Benign diseases could also lead to an elevation of serum CA 19-9 levels. Cholestatic diseases (such as choledocolitiasis) and cholangitis may raise CA 19-9 to very high levels in the absence of malignancy. There are reports of Mirizzi syndrome with CA 19-9 of 21,068 U/mL^18^, making the differential diagnosis with a cholangiocarcinoma very difficult.

## CONCLUSIONS

Clinicians should be aware that there is no recommended use of CA 19-9 as a screening test for pancreatic malignancies. It could be elevated in benign diseases, and in patients with Le A−B−, it is expected to be negative even in the presence of documented malignancies. Avoiding the use of CA 19-9 as a screening test in asymptomatic patients will prevent them from unnecessary, costly, and sometimes invasive diagnostic tests.
